# Evaluation of the Potential of Biofilm Formation of *Bifidobacterium longum* subsp. *infantis* and *Lactobacillus reuteri* as Competitive Biocontrol Agents Against Pathogenic and Food Spoilage Bacteria

**DOI:** 10.3390/microorganisms8020177

**Published:** 2020-01-25

**Authors:** Barbara Speranza, Arcangelo Liso, Vincenzo Russo, Maria Rosaria Corbo

**Affiliations:** 1 Department of the Science of Agriculture, Food and Environment (SAFE), University of Foggia, Via Napoli 25, 71122 Foggia, Italy; barbara.speranza@unifg.it; 2 Department of Medicine and Surgery, University of Foggia, Polo Biomedico, Viale Pinto 1, 71122 Foggia, Italy; 3 Institute of Ophthalmology, Department of Surgery Science, University of Foggia, Viale Pinto, 71122 Foggia, Italy; virus66@alice.it

**Keywords:** probiotic, biofilm, pathogen, spoilage bacteria, active packaging

## Abstract

This study proposes to exploit the in vivo metabolism of two probiotics (*Bifidobacterium longum* subsp. *infantis* and *Lactobacillus reuteri*) which, upon adhesion on a solid surface, form a biofilm able to control the growth of pathogenic and food spoilage bacteria. The results showed that pathogenic cell loads were always lower in presence of biofilm (6.5–7 log CFU/cm^2^) compared to those observed in its absence. For *Escherichia coli* O157:H7, a significant decrease (>1–2 logarithmic cycles) was recorded; for *Listeria monocytogenes*, *Staphylococcus* *aureus,* and *Salmonella enterica*, cell load reductions ranged from 0.5 to 1.5 logarithmic cycles. When tested as active packaging, the biofilm was successfully formed on polypropylene, polyvinyl chloride, greaseproof paper, polyethylene and ceramic; the sessile cellular load ranged from 5.77 log CFU/cm^2^ (grease-proof paper) to 6.94 log CFU/cm^2^ (polyethylene, PE). To test the potential for controlling the growth of spoilage microorganisms in food, soft cheeses were produced, inoculated with *L.* *monocytogenes* and *Pseudomonas fluorescens,* wrapped in PE pellicles with pre-formed biofim, packed both in air and under vacuum, and stored at 4 and 15 °C: an effective effect of biofilms in slowing the decay of the microbiological quality was recorded.

## 1. Introduction

Despite that their use in foods is dated, in the last decades, Lactic Acid Bacteria (LAB) have attracted much attention for their documented beneficial properties and for potential useful applications. Among LAB, several strains are currently claimed as probiotics [1], i.e., live microorganisms that, when administered in adequate amounts, confer a health benefit on the host [2]. According to the consensus statement, there are some bacterial species with a long history of safe use and a well-recognized health effect, such as *Bifidobacterium adolescentis*, *B. animalis, B. bifidum*, *B. breve*, *B. longum*, *Lactobacillus acidophilus*, *L.* *reuteri,* *L.* *casei*, *L.* *fermentum*, *L.* *gasseri,* etc. [2]; some strains, such as *B. longum* subsp. *infantis* and *L.* *reuteri*, are widespread due to the strong evidence of their effect on health [3]. Probiotics are able to colonize, stably or transiently, host mucosal surfaces, including the gut, where they may contribute to host health; the capacity of probiotics to colonize biotic and abiotic surfaces by forming structured communities (i.e., biofilms), could have great potentials for human health and food safety biotechnologies, although this aspect has is in fact barely been explored. It has recently been shown that microbial biofilms may play several “useful” roles such as biodegradation of toxic compounds and pollutants, bioremediation, toxic effluents treatment [4], despite being initially considered only a negative phenomenon. These applications suggest that microbial biofilms could be successfully used for new applications in the biomedical, industrial, food, and environmental field [4]. 

In the biomedical field, for example, a biofilm formed by probiotic microorganisms could be potentially useful to hinder the development of microorganisms responsible for infections, especially those caused by microorganisms of hospitals, typically resistant to common antibiotic treatments. Indeed, it is widely accepted that in the development of direct and airborne transmission of nosocomial infections, the hospital environment (infection reservoir) plays a key role [5]. In fact, it can be anticipated that a probiotic biofilm left to form ad hoc on several surfaces (e.g., toilets, air conditioning systems) could reduce the spread of pathogenic species that may harbor thereon. Other potential applications in the biomedical field could be: preparations used in skin lesions for the healing processes to add antibacterial capacity, the coating of implants and catheters, medical devices applied to the oral cavity which might hinder the growth of bacterial species associated with caries and periodontal disease [6,7,8,9]. 

On the other hand, regarding potential applications in the food industry, biofilms can be used to ensure the hygienic-sanitary safety of food products, as well as an extension of their shelf-life. The formation of biofilms by “useful/probiotic” microorganisms may be stimulated on materials commonly used to package food (plastic films, pellicles, combinations for packaging, paper, etc.) in order to develop an innovative active packaging system. Although the scientific community is very active in the production of research related to the ability of microorganisms to form biofilms, most studies have focused on biofilm formation by pathogens and/or spoilage microorganisms (*Enterobacter*, *Listeria*, *Micrococcus*, *Streptococcus*, *Bacillus* and *Pseudomonas*) [10,11,12,13]. It has been also shown that certain species of LAB are able to form biofilms and some of them are capable of exhibiting antimicrobial activity against pathogenic microorganisms [14,15,16]; some research was conducted on the possibility of using new methods of sanitation, exploiting the principle of biological competition using probiotic products [17], but this aspect needs to be explored further. In a previous study, we have described the optimization of the production of a probiotic biofilm through intermediate steps by fixing some valuable key points about the probiotics’ ability to adhere to surfaces and to form biofilms [18]. These results were used to file a patent covering the use of probiotic biofilms as a means to control pathogen growth [19]. Even if some studies in literature present the use of LAB (mainly lactobacilli) biofilms to control pathogen growth in food and superficies [20,21,22,23,24,25,26,27], most of them propose the use of bio-surfactants and compounds with antimicrobial activity produced in greater quantities by lactobacilli when growing in sessile form. Indeed, our study proposes a probiotic biofilm that exploits the in vivo metabolism of two selected probiotic strains able to adhere rapidly on abiotic surfaces, and not the substances secreted by them and subsequently recovered and used, as in the prior art. To the best of our knowledge, only one study has previously proposed a similar approach evaluating the use of potential probiotic LAB (isolated from Brazilian′s foods) biofilms to control *Listeria monocytogenes*, *Salmonella* Typhimurium, and *Escherichia coli* O157:H7 biofilms formation and suggesting that LAB strains can be excellent candidates to form protective biofilms to be used as biocontroller of contamination into the food chain [28]. 

Besides the use as an innovative active packaging to ensure the safety of food products, as well as an extension of their shelf-life, the proposed probiotic biofilm formed ad hoc on medical devices (catheters, implants, braces, bite blocks or condoms) and on bathrooms’ surfaces (sink, bidet, toilet bowl, water closet or piece of furniture) could be considered a tool against colonizing strains, since these surfaces are often implicated in nosocomial infections. Our proposal could lead to the development of a useful means to control the growth of pathogenic and spoilage bacteria for industrial and medical applications. In the following, some specific applications of the developed probiotic biofilm are described, focusing on two different aspects: 1) effect of probiotic biofilms on pathogen sessile growth; 2) application as potential active packaging.

## 2. Materials and Methods 

### 2.1. Effect of Probiotic Biofilms on Pathogen Sessile Growth 

#### 2.1.1. Surfaces and Microorganisms

Polycarbonate resin (Lexan, Fedele s.r.l., Rome, Italy) was the surface chosen for the adhesion experiments. Before each experiment, the chips (2.5 × 5.0 × 0.05 cm) were prepared by washing in acetone for a minimum of 30 min, rinsing in distilled water, and then soaking in 1 N NaOH for 1 h. After a final rinse in distilled water, the chips were allowed to air dry. This cleansing procedure was required to remove fingerprints, oils grease, and other soils that may have been on the materials. The cleaned chips were finally autoclaved at 121 °C for 15 min prior to use. 

The probiotic strains used for this study were *Bifidobacterium longum* subsp. *infantis* DSM20088 and *Lactobacillus reuteri* DSM20016, both purchased from Leibniz-Institut DSMZ (Deutsche Sammlung von Mikroorganismen und Zellkulturen) and stored at −20 °C in MRS broth (Oxoid, Milan, Italy). 

Before each assay, they were grown in their optimal media at their optimal conditions, until late exponential phase was attained; namely, MRS broth added with cysteine 0.05% (w/v) (Sigma-Aldrich, Milan, Italy) incubated at 37 °C for 24 to 48 h, under anaerobic conditions, and MRS broth (Oxoid) incubated at 30 °C for 24 to 48 h, under anaerobic conditions, were used for *B. infantis* DSM20088 and *L.* *reuteri* DSM20016, respectively. 

Cells cultures were successively harvested by centrifugation for 10 min at 4500 rpm (4 °C) and the pellets were washed twice with sterile saline solution (0.9% NaCl) at 4 °C and finally resuspended in the same solution at a cell concentration of 1 × 10^8^ CFU/mL. 

As pathogen targets were chosen, four strains belonging to the Culture Collection of the Laboratory of Predictive Microbiology (Department of the Science of Agriculture, Food and Environment, Foggia University), and microorganisms with the media and growth conditions used, have been listed in Table 1. The organisms were transferred to fresh Nutrient Agar (NA, Oxoid) periodically to maintain viability and, prior to use, they were activated by two successive 24-h transfers of cells in Nutrient broth (NB, Oxoid) at 37 °C. Inocula for experiments were prepared by centrifugation of the 24-h microbial cultures at 3000× *g* for 15 min at 4 °C. After centrifugation, the obtained pellets were resuspended in sterile saline solution at 4 °C to obtain approximately 10^8^ CFU/mL for each microorganism. 

#### 2.1.2. Experiment

Biofilm formation was favoured by simultaneously inoculating the cocktail of identified probiotics (*B. infantis* DSM20088 and *L.* *reuteri* DSM20016, about ~10^8^ CFU/mL) and the pathogenic target (~10^7^ CFU/mL) on polycarbonate surfaces (Lexan^®^ tiles, 25 mm × 75 mm, 0.5 mm thick) left at room temperature (20 °C) for 2 h. After this time interval, the tiles were transferred to aliquots of peptone water (1% bacteriological peptone) and incubated at 15 °C for 48 h [18,19]. Specifically, for each pathogen, two samples were prepared: an ACTIVE sample (ACT), containing a chip where probiotics were left to form biofilm; a CONTROL sample (CNT), containing a chip without probiotics. The pathogen sessile cell load was determined after 0, 4, 24, 30 and 48 h after inoculation. At these times, chips were aseptically removed and rinsed with sterile distilled water, in order to eliminate the unattached cells. As suggested in literature [29], sessile cells were detached from chips in a sterile test tube containing 45 mL of sterile saline with a 20 Hz “Vibra Cell” sonicator (SONICS, Newcastle, Conn., USA) for 3 min. Viable and cultivable cells were enumerated by serial dilutions in 0.9% NaCl solution and plating on appropriate media (Table 1). 

### 2.2. Application as Potential Active Packaging 

#### 2.2.1. Probiotic Biofilm Formation on Different Materials

The materials assayed were polypropylene (PP), polyvinyl chloride (PVC), greaseproof paper (GP), waxed paper (WP), polyethylene (PE) and ceramic; all materials were cut in rectangles of 2.5 × 5.0 cm and cleaned by immersion in ethanol. Each individual chip was well rinsed with ultrapure water and dried at room temperature. Probiotic biofilms were left to form for 96 h by simultaneously inoculating the cocktail of probiotics (*B. infantis* DSM20088 and *L.* *reuteri* DSM20016, about ~10^8^ CFU/mL) on chips of different materials, left at room temperature (20 °C) for 2 h. After this time interval, the chips were transferred to aliquots of peptone water (1% bacteriological peptone) and incubated at 15 °C for 96 h [19].

Biofilm cells were enumerated at 2, 24, and 96 h after inoculation. At these times, chips were aseptically removed and rinsed with sterile distilled water, in order to eliminate the unattached cells. Sessile cells were detached from chips in a sterile test tube containing 45 mL of sterile saline with a “Vibra Cell” sonicator (SONICS, Newcastle, Conn., USA) at 20 kHz for 3 min. Viable and cultivable cells were enumerated by serial dilutions in 0.9% NaCl solution and plating on MRS Agar (Oxoid). 

#### 2.2.2. Challenge Tests

Inoculations for experiments were prepared by centrifugation of the 24-h microbial cultures in an ALC 4239R centrifuge (ALC, Milan, Italy) at 3000× *g* for 15 min at 4 °C. For the inoculations of challenge tests, after centrifugation the pellets were resuspended in sterile isotonic solution (0.9% NaCl) at a temperature of 4 °C and serial dilutions were made to obtain approximately 10^4^ CFU/mL for each microorganism. For biofilm formation, the probiotic pellet was resuspended in sterile isotonic solution at a temperature of 4 °C and used on polyethylene films to form experimental pellicles with pre-formed probiotic biofilm (EXP).

Miniature soft cheeses were made using pasteurized, whole and homogenized milk, purchased in a local market. The milk had the following characteristics: lactose 5.0%, protein 3.2%, fat 3.6%, pH 6.6. The cheeses were produced using a domestic cheese-maker (“Casaro”, Philips, Milan, Italy) by pouring the milk into the single-wall cheese-maker vessel and heating to 85 °C. As soon as the temperature reached 85 °C (after a few minutes), 4 g/L of sodium chloride was added and the salted milk was immediately left to cool to 30 °C. Renneting was performed with 3 mL/L of liquid calf rennet (concentrate extract of Liquid Rennet, CHR. Hansen s.p.a., Milan, Italy). After coagulation and curd strengthening (approximately 40 min), the curd was cut and the whey discarded. Finally, miniature soft cheeses of a round shape (25 g, 6 cm diameter) and regular smooth surfaces were made by hand and placed in sterile boxes fitted with a grid to facilitate whey draining. The boxes were kept at room temperature for 6 h until packaging.

To test the potential for probiotic biofilms to control the growth of microorganisms in soft cheese, they were inoculated with *L.* *monocytogenes* (challenge test A) and *Ps. fluorescens* (challenge test B). The inoculation (about 10^2^ CFU/g) was carried out in the most homogeneous way possible, spreading 0.2 mL of the prepared microbial suspension across the entire surface of miniature cheese by means of a sterile spatula. After inoculation, all cheeses were wrapped in polyethylene films (EXP) and packed in high-barrier plastic bags (Nylon/Polyethylene, 102 µm (Tecnovac, San Paolo D’Argon, Bergamo, Italy)) by means of S100-Tecnovac equipment. Control batches were prepared by wrapping cheeses in pellicles without pre-formed biofilm (CNT). All samples were packaged in air and under vacuum. During the storage at 4 and 15 °C for 28 and 14 days, respectively, microbiological analyses, determination of pH and measurements of *a_w_* were made, details of which are given below. 

#### 2.2.3. Microbiological, Chemico-Physical and Sensorial Analyses 

For microbiological analyses, mini-cheeses (25 g) were diluted with 225 mL of 0.1% peptone water with salt (0.9% NaCl) in a Stomacher bag (Seward, London, England) and homogenized for 1 min in a Stomacher Lab Blender 400 (Seward). Serial dilutions of cheese homogenates were plated on the surface of the appropriate media in Petri dishes. The media and the conditions used were: *Listeria* selective agar base (Oxoid) plus *Listeria* selective supplement-Oxoid formulation, incubated at 37 °C for 48 h, for *L.* *monocytogenes*; *Pseudomonas* Agar Base (Oxoid) plus *Pseudomonas* CFC Supplement, incubated at 25 °C for 48 h, for *Ps. fluorescens*; MRS agar (Oxoid), incubated at 30 °C for 4 days under anaerobiosis, for mesophilic lactobacilli.

For each batch, the measurement of pH was performed twice on the first homogenized dilution of the cheese samples during storage with a Crison pH meter model micro pH 2001 (Crison). *a_w_* was measured by a hygrometer AQUALAB CX-2 (Decagon Device, Pullman, WA, USA). 

During the storage at 4 and 15 °C, a sensory evaluation was also performed: the panel consisted of 15 panelists aged between 22 and 38 years (students and researchers of the Department of the Science of Agriculture, Food and Environment (SAFE), University of Foggia). Using a scale ranging from 0 to 10 (where 10 stands for the most attractive attributes and 0 for the absolutely unpleasant attributes), the sensorial overall quality of the samples was determined by evaluating colour, odour, texture and overall acceptability. During the test sessions, cheese samples were coded by a letter and presented individually to each panelist in plastic cups covered with a lid in random order. Sensory evaluation was conducted in individual booths under controlled conditions of light (white light), temperature (20 ± 2 °C), and humidity (70% to 85%). 

#### 2.2.4. Statistical Analyses

All experiments were performed twice with the analyses conducted twice. 

Results about the effect of probiotic biofilms on pathogen sessile growth were expressed as log CFU/cm^2^, presented as the average of replicates (*n* = 4) and analyzed through the Student’s t-test (*p* < 0.05).

To highlight the effectiveness of probiotic biofilm, for each time of analysis the pathogen sessile data were expressed as follows:

Biofilm Efficacy = CNT − ACT

where CNT and ACT were pathogen cell numbers (log CFU/cm^2^) in the control (without probiotic biofilm) and in the active sample (with probiotic biofilm). These differences were analysed through one-way ANOVA and Tukey’s test as the post-hoc comparison test (*p* < 0.05). Results about biofilm formation on different materials were expressed as log CFU/cm^2^, presented as the average of replicates (*n* = 4) and analyzed through one-way ANOVA and Tukey’s test as the post-hoc comparison test (*p* < 0.05).

The microbiological data collected during the challenge tests were expressed as the average of two replicates and the obtained mean values (one for experiment) were modelled according to the Gompertz equation modified by Zwietering et al. [30]:

(1) $$y=k+A*exp\left\{-exp\left[\left(\mu_{max}*e/A\right)*\left(\lambda -t\right)+1\right]\right\},$$

where *y* is the concentration of the microorganism (Log CFU/ g), *k* is the initial level of the dependent variable to be modelled, *A* is the difference between the decimal logarithm of the initial value of cell concentration and the decimal logarithm of maximum bacteria growth attained at the stationary phase (Log CFU/g), µ*_max_* is the maximal growth rate (1/day), λ the lag time (day) and *t* the time.

Following Castillejo Rodriguez et al. [31], the sanitary risk time for the growth of *L.* *monocytogenes* in our samples was determined as the time (in days) that it took to observe an increase of 2 Log CFU/g of the count of this microorganism in food as follow:

sanitary risk time [SRT] = 2/µ,(2)

where µ is the maximal growth rate.

For the growth of *Ps. fluorescens*, the maximum acceleration of microbial growth (dy^2^/dt^2^ (day)), known as stability time, was also estimated with the Gompertz equation, following Riva et al. [32].

To determine whether significant differences (*p* < 0.05) existed among the parameters calculated by using the Gompertz equation, a one-way analysis of variance (ANOVA), followed by Tukey’s test, was conducted.

Modeling was performed through the software Statistica for Windows version 10.0 (Statsoft, Tulsa, OK, USA).

## 3. Results and Discussion

### 3.1. Effect of Probiotic Biofilms on Pathogen Sessile Growth

In order to evaluate the effect of probiotic biofilms on the development of pathogenic microorganisms, evidence was provided on the growth in sessile form of *L.* *monocytogenes, E. coli* O157:H7, *St. aureus* and *S. enterica*. Table 2 shows the cellular loads in sessile form relating to the targets studied; the data analysis shows how the pathogens studied were able to develop in all samples, even if they exhibited a wide range in their ability to colonize the surface, with the highest initial adhesion recovered for *S. enterica* (about 6 log CFU/cm^2^) against the lowest one (about 4 log CFU/cm^2^) recovered for *L.* *monocytogenes*. However, cellular loads were always lower in ACT samples (presence of probiotic biofilm, about 6.5–7 log CFU/cm^2^) compared to the CNT samples (absence of probiotic biofilm), highlighting that the studied biofilm was able to control the growth of all inoculated pathogenic targets. To quantify the effectiveness of probiotic biofilms in slowing down the pathogens’ adhesion, for each time of analysis the difference between the cellular loads recovered in CNT and ACT samples was calculated. As it can be inferred from Table 2, for *E. coli* O157:H7, there was a significant decrease in cell load compared to control of more than 1 and 2 logarithmic cycles after 4 and 48 h of incubation, respectively, and the biofilm efficacy increased over time. Similar results were observed for *St. aureus*. On the contrary, for *L.* *monocytogenes* the effectiveness of probiotic biofilm was maximum after 4 h (1.43 ± 0.28), but it decreased over time; this loss of efficacy was also recorded for *S. enterica*, with cell load reductions ranging from 1 to 0.2 logarithmic cycles after 24 and 48 h, respectively. As expected, biofilms were odorless and invisible to the naked eye. The idea to use probiotics into the prevention of infections and other diseases has already been proposed [7], and is also stimulated by the need of new alternative intervention strategies to combat bacteria pathogenesis due to the increasing evidence of antibiotics resistance of many pathogens. Abdelhamid et al. [33] observed that cell-free preparations of different probiotics belonging to *Lactobacillus* and *Bifidobacterium* species were able to reduce the growth of *E. coli*, whereas Kaboosi [34] showed that probiotics from yogurts had antibacterial effects against Gram negative bacteria such as *E. coli*, *Salmonella* Typhi and *Ps. aeruginosa*, and Gram positive bacteria such as *S. aureus*. Similarly, Tejero-Sariñena et al. [35] found that 15 strains of probiotics had antibacterial properties against gram negative *Salmonella* Typhimurium and *Clostridium difficile*. However, most of these studies propose the use of compounds (mainly bio-surfactants) with antimicrobial activity produced by probiotics, and contained in their cell-free supernatants [20,21,22,23,24,25,26,27]; on the contrary, this study proposes the use of a probiotic biofilm that exploits the in vivo metabolism of two bacterial strains (*Lactobacillus* and *Bifidobacterium*) adhering on abiotic surfaces and not the substances secreted by probiotics and subsequently recovered and used. In 2014 Schobitz et al. [36] proposed a biocontroller consisting of the thermally treated fermentate (TTF) from two *Carnobacterium maltaromaticum* strains (ATCC PTA 9380 and ATCC PTA 9381), a strain of *Enterococcus mundtii* (ATCC PTA 9382), plus nisin at a concentration of 1000 IU/mL, with all these components entrapped in an alginate matrix supported by a mesh-type fabric. The strains used in our study are different, and no bacteriocin or polymer is used, but the proposed probiotic biofilm should be formed on different surfaces chosen according the purpose (an active packaging and/or a medical device). Moreover, our solution, thanks to the maintenance of a continuous metabolism, should ensure an uninterrupted and stronger activity of the active substances (mainly bacteriocins and/or other LAB-produced antimicrobial compounds such as hydrogen peroxide, carbon dioxide, diacetyl, organic acids), being the same in loco produced [37]. Similar to our study, Gomez et al. [28] used in situ biofilms formed by potential probiotic LAB strains isolated from Brazilian′s foods (*Lactococcus lactis* VB69, *L.* *lactis* VB94, *Lactobacillus sakei* MBSa1, *Lactobacillus curvatus* MBSa3, *L.* *lactis* 368, *Lactobacillus helveticus* 354, *Lactobacillus casei* 40, and *Weissela viridescens* 113) to inhibit pathogenic growth: they found the total inhibition in pathogens *E. coli* O157:H7, *L.* *monocytogenes* and *Salmonella* Typhimurium biofilm formation, in 24, 48, and 72 h of exposure using *L.* *lactis* 368, *Lactobacillus curvatus* MBSa3 and *Lactobacillus sakei* MBSa1. For the other strains, the inhibition was time-dependent and varied according to the strain and target pathogen; for *L.* *monocytogenes,* reductions ranged from 4- to 7-log units over 24 and 48 h, and the inhibition was observed only within the first 24–48 h, after which the pathogen was able to grow. In *Salmonella* Typhimurium and *E. coli* O157:H7 experiments, sessile cells were not detected during 24 h of incubation in the presence of most LAB tested; during 48 and 72 h, reductions between 5 and 3 log for *E. coli* O157:H7 and 4 log for *Salmonella* Typhimurium were achieved.

### 3.2. Application as Potential Active Packaging

Once ascertained the effects on pathogens growth, the research focused on the formation of the probiotic biofilm on different packaging materials, in order to individuate an innovative packaging system.

The results obtained are shown in Table 3; after only 2 h, the probiotic biofilm was successfully formed on all tested materials, except for waxed paper. The sessile cellular load ranged from 5.77 log CFU/cm^2^ (grease-proof paper) to 6.94 log CFU/cm^2^ (polyethylene). After 96 h, polyethylene and ceramic resulted the materials on which the highest adhesion was recorded (6.54 log CFU/cm^2^). In general, any surface (plastic, rubber, glass, metal, paper, cement, stainless steel or wood, or food products themselves) are vulnerable to biofilm development and each biofilm is different, thus suggesting that every situation should be analysed individually and specifically [38].

Once individuated in polyethylene (PE) the material able to ensure the greatest adhesion of probiotics, in a second step, the attention was focused only on this material and it was used to test the potential for probiotic biofilms to control the growth of microorganisms in soft cheeses. The products were inoculated with *L.* *monocytogenes* (challenge test A) and *Ps. fluorescens* (challenge test B), wrapped in PE pellicles with pre-formed probiotic biofim, packed both in air and under vacuum, and stored at 4 and 15 °C. These model bacteria were chosen as main representatives of pathogen and spoilage bacteria naturally contaminating soft cheese [39,40].

**Figure 1:**
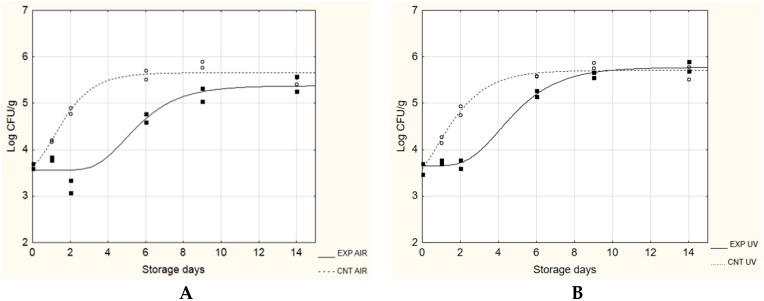
Evolution of *L. monocytogenes* during the challenge test at 15 °C. EXP, cheeses stored with probiotic biofilms; CNT, cheeses stored without probiotic biofilm. (**A**), AIR packaging; (**B**), under vacuum packaging (UV).

At 4 °C, the cellular load of *L.* *monocytogenes* remained lower than 3 log CFU/g for the entire observation period (28 days), regardless the presence of the probiotic biofilm or the packaging. On the other hand, at 15 °C (simulated thermal abuse), the λ length was always longer in samples containing probiotic biofilms (EXP samples), if compared to CNT samples (without probiotic biofilms) (Table 4): its value increased from 0.04 to 3.37 days (in air packaging, Figure 1A) and from 0.00 to 2.40 days (under vacuum, Figure 1B). The growth rate (µ_max_) was also influenced by the presence of probiotic biofilms, recording a decrease from about 0.7 to 0.4 Log(CFU/g)/day, in both packaging conditions. The maximum cell load reached in the stationary phase (A + N_0_) was not influenced, reaching approximately 5.6–5.7 log CFU/g, regardless of the presence or absence of probiotic biofilms. The cellular load of lactic bacteria (LAB) was also monitored, as well as pH and water activity. At 4 °C, the initial LAB count was 5.75 ± 0.18 log CFU/g in the control samples against 8.32 ± 0.20 log CFU/g in the experimental cheeses; after 28 days, there were no statistically significant differences between the samples (regardless of the presence of probiotic biofilms and the type of packaging), recording cellular loads between 7 and 8 log CFU/g (data not shown).

Additionally, for the pH, no significant differences between the samples were observed; this parameter decreased from 5.31–5.39 to 4.70–4.84 at the end of storage. In all samples, the value of water activity remained constant (0.99–1.00) for the entire duration of the experimentation (data not shown). Similar results were observed at 15 °C.

**Figure 2:**
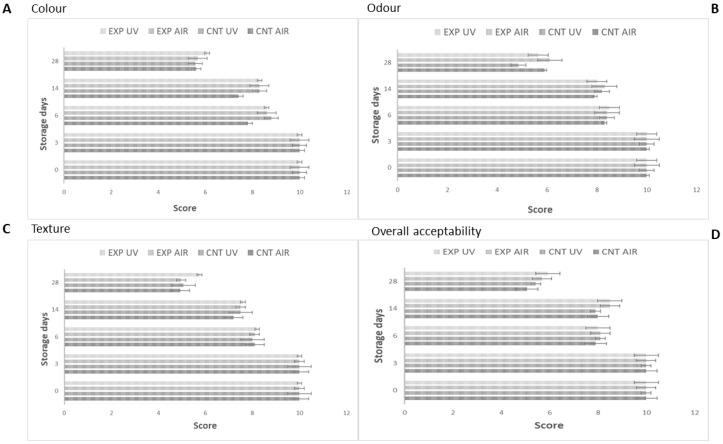
Sensorial scores for colour (**A**), odour (**B**), texture (**C**) and overall acceptability (**D**) of cheeses inoculated with *L. monocytogenes* stored at 4 °C. Mean values ± standard deviation. EXP, cheeses stored with probiotic biofilms; CNT, cheeses stored without probiotic biofilm.

During the experimentation, both at 4 and 15 °C, a gradual decrease of the score from 10 to about 5.5–6 was recorded (end of storage), regardless of the presence of probiotic biofilms and the type of packaging applied, showing that the probiotic microorganisms had no impact on the sensory characteristics of cheeses; as an example, Figure 2 shows the sensorial scores for colour, odour, texture and overall acceptability of cheeses recovered during storage at 4 °C.

**Figure 3:**
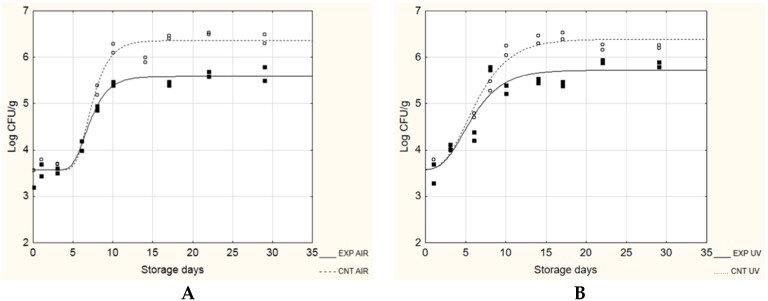
Evolution of *Ps. fluorescens* during the challenge test at 4 °C. EXP, cheeses stored with probiotic biofilms; CNT, cheeses stored without probiotic biofilm. (**A**), AIR packaging; (**B**), under vacuum packaging (UV).

**Figure 4:**
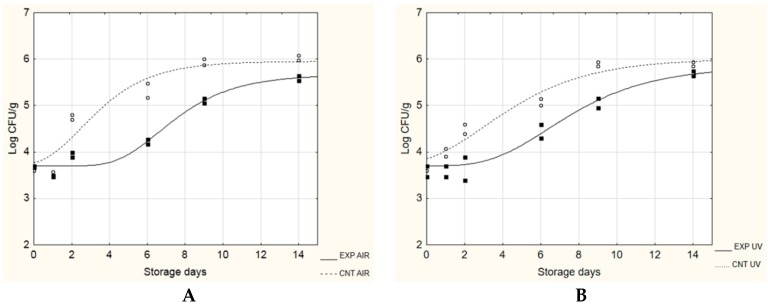
Evolution of *Ps. fluorescens* during the challenge test at 15 °C. EXP, cheeses stored with probiotic biofilms; CNT, cheeses stored without probiotic biofilm. (**A**), AIR packaging; (**B**), under vacuum packaging (UV).

Figure 3; Figure 4 show the evolution of *Ps. fluorescens* during the growth on EXP and CNT cheeses stored at 4 and 15 °C, respectively. The target microorganism was able to grow under all tested conditions, regardless of the presence of probiotic biofilms and the type of packaging. At 4 °C (Figure 3), the presence of the probiotic biofilm was able to influence the maximum cellular load reached in the stationary phase (A + N_0_), which was significantly lower in the EXP samples (5.59–5.72 log CFU/ g) compared to the CNT samples (6.36–6.39 log CFU/g). No influence was observed about the λ length and the maximum growth rate (µ_max_).

During storage at 15 °C (Figure 4), the presence of probiotic biofilms significantly slowed the growth of the target microorganism: λ increased from 0.54 to 4.40 days and from 0.01 to 3.30 days, in air and vacuum packaging, respectively. The maximum growth rate and the maximum cell load reached in the stationary phase were also lower in the EXP samples (probiotic biofilms) than the control samples, regardless of the packaging applied.

At both 4 and 15 °C, data on LAB, pH and water activity were similar to those observed in the challenge test with *L.* *monocytogenes* (data not shown). Results of sensory analyses confirmed that the probiotic microorganisms had no impact on the organoleptic characteristics of cheeses (data not shown).

To highlight the effectiveness of probiotic biofilms to slow the decay of the microbiological quality of soft cheeses at 15 °C, Table 4 shows the kinetic parameters of Gompertz equation accompained by two other parameters (TRS and stability time). In a well-known study on the growth of *L.* *monocytogenes* in food, Castillejo Rodriguez et al. [30] have proposed the sanitary risk time (TRS) for this pathogen as the time required (in days) to observe an increase of 2 log CFU/g in its count, considering that, under normal conditions, such a microorganism is present in foods in very low concentrations. As can be seen, for soft cheeses wrapped in probiotic biofilms and packaged both in air and under vacuum, the TRS was equal to 4.40–4.60 days; on the contrary, the same methods of packaging, applied to the control samples, allowed *L.* *monocytogenes* to reach risky cell counts in shorter times (2.88–2.95 days) (*p* < 0.05).

For the tests conducted with *Ps. fluorescens* at 15 °C, Table 4 also shows the stability time [31,41] which represents the maximum acceleration of microbial growth and indicates how long the product remains stable: after this time, an irreversible decay of the product begins. This parameter is generally used as an alternative to shelf life: the underlying principle implies that microbial degradation has to show a rate of the same order of magnitude as at the shelf life zero time. This condition is no longer met when microbial growth attains its maximum acceleration, because beyond such a threshold the system undergoes very fast changes with a rapid loss of the generally accepted safety or quality requirements. This principle seems more reliable than the current practice that defines food stability according to the ratio between attained and starting microbial population levels. The stability time increased by more than 3 and 4 days, in vacuum packaging and in air, respectively, highlighting the effectiveness of biofilms in slowing the decay of the microbiological quality of soft cheese.

Regarding the inhibitory effect of LAB against *L.* *monocytogenes*, some studies have already explored the possibility to use a preformed biofilm to inhibit the pathogen growth [15,16,18,42]. Namely, Guerrieri et al. [15] showed the potential of a *Lactobacillus plantarum* strain to reduce the pathogen growth over a 10-day period (about 4-log reduction). Mariani et al. [42] used the native biofilm microflora of wooden cheese ripening shelves to achieve a 1- to 2-log reduction over a 12-day period. In previous studies, we have evaluated the use of LAB biofilms as a means to control the growth of *L.* *monocytogenes* in soft cheeses [16] and in laboratory media [18], finding that sessile LAB biofilms were able to delay the growth of *L.* *monocytogenes.* An anti-listerial activity was also observed by Léonard et al. [43] during their studies on biopolymeric matrices based on alginate and alginate-caseinate (an aqueous two-phase system) entrapping *Lactococcus lactis* subsp. *lactis* LAB3 cells and by Barbosa et al. [44] who entrapped *Lactobacillus curvatus* in calcium alginate: the effect against the pathogen was correlated to antimicrobial metabolites of proteinaceous nature.

## 4. Conclusions

This study has explored whether probiotic bacteria able to adhere on different surfaces (i.e., packaging materials, ceramic, plastic, paper, polymers, etc.) could be used as new biotechnological solutions for industrial applications by biocontrolling the growth of pathogenic and spoilage bacteria.

The results obtained have shown the studied biofilm was able to delay the growth of some pathogenic targets; in fact, cellular pathogenic loads were always lower in presence of probiotic biofilm compared to its absence. For *E. coli* O157:H7, a significant cell load decrease (>1–2 logarithmic cycles) was recorded, whereas for *L.* *monocytogenes*, *St. aureus* and *S. enterica*, cell load reductions ranged from 0.5 to 1.5 logarithmic cycles.

After only 2 h, the probiotic biofilm was successfully formed on polypropylene, polyvinyl chloride, greaseproof paper, polyethylene and ceramic, with polyethylene and ceramic resultingly being the material with the highest adhesion (6.54 log CFU/cm^2^). When testing as a tool to control the growth of microorganisms in soft cheeses, the results highlighted the effectiveness of biofilms in slowing the growth of *L.* *monocytogenes* by prolonging their microbiological stability at 15 °C by more than 3 and 4 days.

The results obtained suggest that the developed probiotic lactic acid bacteria biofilms have a good potential to be used as biocontrol agents against pathogenic and food spoilage bacteria through exclusion mechanisms: however, the mechanisms responsible for the inhibition have to be deeply investigated.

## 5. Patents

B.S., A.L. and M.R.C. applied for a patent covering the use of probiotic biofilms as a mean to control pathogens growth: Method for producing microbial probiotic biofilms and uses thereof (WO2017203440). International Application No.: PCT/IB2017/053055. National Application No.: P1287IT.

## Figures and Tables

**Table 1:** Pathogen strains used in the study with the indication of their source and optimal media and growth conditions adopted.

Strains	Source	Optimal Media and Growth Conditions
Listeria monocytogenes *	Culture Collection of the Laboratory of Predictive Microbiology, SAFE, University of Foggia	Listeria selective agar base (Oxoid) plus Listeria selective supplement-Oxoid formulation, incubated at 37 °C for 48 h
*Escherichia coli* O157:H7	CECT 4267	Sorbitol MacConkey Agar (Oxoid), incubated at 37 °C for 24 h
*Staphylococcus aureus*	ATCC 25923	Baird-Parker Agar Base (Oxoid) plus Egg Yolk Tellurite Emulsion, incubated at 37 °C for 24 h
*Salmonella enterica*	ATCC 35664	Chromatic Salmonella Agar (Liofilchem, Roseto degli Abruzzi, Teramo, Italy), incubated at 37 °C for 24 h

* The strain was isolated from fish products and identified by sequencing the 16SrDNA.

**Table 2:** Cellular loads (Log CFU/cm^2^) recovered for *Listeria monocytogenes*, *Escherichia coli* O157:H7, *Staphylococcus aureus* and *Salmonella enterica* during their sessile growth with (ACTIVE, ACT) or without (CONTROL, CNT) probiotic biofilms.

	*L. monocytogenes*		
**Time (h)**	**CNT**	**ACT**	** Biofilm Efficacy
0	4.21 ± 0.01 ^A,^*	3.46 ± 0.12 ^B^	0.75 ± 0.17 ^a,^***
4	4.82 ± 0.16 ^A^	3.39 ± 0.20 ^B^	1.43 ± 0.28 ^b^
24	4.83 ± 0.13 ^A^	4.10 ± 0.10 ^B^	0.73 ± 0.14 ^a^
30	5.18 ± 0.25 ^A^	4.31 ± 0.11 ^B^	0.87 ± 0.16 ^a^
48	4.91 ± 0.01 ^A^	4.23 ± 0.10 ^B^	0.68 ± 0.14 ^a^
	*E. coli* O157:H7		
**Time (h)**	**CNT**	**ACT**	**Biofilm Efficacy**
0	5.49 ± 0.01 ^A^	5.20 ± 0.25 ^A^	0.29 ± 0.35 ^a^
4	5.43 ± 0.14 ^A^	4.19 ± 0.22 ^B^	1.24 ± 0.31 ^b^
24	5.56 ± 0.41 ^A^	4.10 ± 0.01 ^B^	1.46 ± 0.01 ^b^
30	6.13 ± 0.30 ^A^	3.82 ± 0.01 ^B^	2.31 ± 0.01 ^c^
48	6.00 ± 0.25 ^A^	3.80 ± 0.20 ^B^	2.20 ± 0.28 ^c^
	*St. aureus*		
**Time (h)**	**CNT**	**ACT**	**Biofilm Efficacy**
0	5.07 ± 0.20 ^A^	4.88 ± 0.01 ^A^	0.19 ± 0.01 ^a,b^
4	5.02 ± 0.03 ^A^	4.70 ± 0.33 ^A^	0.32 ± 0.47 ^b,c^
24	5.57 ± 0.14 ^A^	4.89 ± 0.19 ^B^	0.68 ± 0.27 ^b,c^
30	5.16 ± 0.01 ^A^	3.86 ± 0.22 ^B^	1.30 ± 0.31 ^c,d^
48	5.16 ± 0.30 ^A^	3.71 ± 0.05 ^B^	1.45 ± 0.07 ^d^
	*Salmonella enterica*		
**Time (h)**	**CNT**	**ACT**	**Biofilm Efficacy**
0	5.94 ± 0.10 ^A^	4.37± 0.10 ^B^	1.57 ± 0.14 ^a^
4	5.38 ± 0.10 ^A^	4.47 ± 0.16 ^B^	0.91 ± 0.23 ^b^
24	5.53 ± 0.15 ^A^	4.54 ± 0.13 ^B^	0.99 ± 0.18 ^b^
30	5.35 ± 0.23 ^A^	4.88 ± 0.05 ^B^	0.47 ± 0.07 ^c^
48	4.98 ± 0.30 ^A^	4.77 ± 0.00 ^B^	0.21 ± 0.00 ^c^

* A, B, Values in the same lines with different letters are significantly different (Student′s t-test) (*p* < 0.05). ** Biofilm Efficacy = CNT–ACT. *** a, b, c, d, Values in the same columns with different letters are significantly different (one-way ANOVA and Tukey′s test) (*p* < 0.05).

**Table 3:** Cellular probiotic load in sessile form (log CFU/cm^2^) observed on common packaging materials used in the food industry and on ceramic.

Materials	Cellular Probiotic Load in Sessile Form (log CFU/cm^2^)		
	**2 h**	**24 h**	**96 h**
Polypropylene (PP)	6.64 ± 0.00 ^A^	6.14 ± 0.48 ^A^	5.88 ± 0.23 ^A^
Polyvinyl chloride (PVC)	6.54 ± 0.14 ^A^	5.65 ± 0.10 ^A^	5.87 ± 0.30 ^A^
Greaseproof paper (GP)	5.77 ± 0.23 ^B^	5.24 ± 0.15 ^B^	5.25 ± 0.06 ^B^
Waxed paper (WP)	No adhesion	4.61 ± 0.22 ^C^	4.53 ± 0.13 ^C^
Polyethylene (PE)	6.94 ± 0.00 ^A^	6.03 ± 0.38 ^A^	6.54 ± 0.14 ^D^
Ceramic	6.86 ± 0.20 ^A^	6.24 ± 0.23 ^A^	6.54 ± 0.14 ^D^

A, B, C, Values in the same columns with different letters are significantly different (one-way ANOVA and Tukey′s test) (*p* < 0.05).

**Table 4:** Kinetic parameters calculated by fitting Gompertz equation to the experimental data by *L. monocytogenes* and *Ps. fluorescens* during their growth in soft cheeses with (EXP) or without (CNT) probiotic biofilms, packed in AIR o under vacuum (UV) and stored at 15 °C. (A + No) is the maximum bacterial load attained at the stationary phase, μ_max_ is the maximal growth rate, λ is the lag time, TRS is the sanitary risk time, ST (stability time) is the maximum acceleration of microbial growth.

*L. monocytogenes*				
	**A + No** **[Log CFU/g]**	**µmax** **[Log(CFU/g)/day]**	**λ** **[day]**	TRS * **[day]**
CNT AIR	5.66 ± 0.31 ^A^	0.69 ± 0.14 ^A^	0.04 ± 0.67 ^A^	2.88
EXP AIR	5.37 ± 0.20 ^A^	0.43 ± 0.19 ^A^	3.37 ± 1.06 ^B^	4.62
CNT UV	5.94 ± 0.85 ^A^	0.68 ± 0.09 ^A^	0.00 ± 0.00 ^A^	2.95
EXP UV	5.39 ± 0.11 ^A^	0.47 ± 0.09 ^A^	2.40 ± 0.72 ^B^	4.30
*Ps. fluorescens*				
	**A + No** **[Log CFU/g]**	**µmax** **[Log(CFU/g)/day]**	**λ** **[day]**	ST ** **[day]**
CNT AIR	5.95 ± 0.33 ^A^	0.41 ± 0.23 ^A^	0.54 ± 0.61 ^A^	2.54
EXP AIR	5.66 ± 0.18 ^A^	0.33± 0.09 ^A^	4.40 ± 0.74 ^B^	6.59
CNT UV	6.00 ± 0.19 ^A^	0.28 ± 0.05 ^A^	0.00 ± 0.50 ^A^	3.03
EXP UV	5.84 ± 0.25 ^A^	0.26 ± 0.05 ^A^	3.33 ± 0.76 ^B^	6.35

A, B, Values in the same columns with different letters are significantly different (one-way ANOVA and Tukey′s test) (*p* < 0.05). *, TRS, sanitary risk time, i.e., the time required (in days) to observe an increase of 2 log CFU/g in *L. monocytogenes* count [30]. **, stability time, i.e., the maximum acceleration of microbial growth [dy^2^/dt^2^ (day)] [31].
